# A facile calcination conversion of groundwater treatment sludge (GTS) as magnetic adsorbent for oxytetracycline adsorption

**DOI:** 10.1038/s41598-021-84231-8

**Published:** 2021-03-05

**Authors:** Asghar Khan, Yang Huo, Zhan Qu, Yanwen Liu, Zhihua Wang, Yu Chen, Mingxin Huo

**Affiliations:** 1 Science and Technology Innovation Center for Municipal Wastewater Treatment and Water Quality Protection, Northeast Normal University, Changchun, 130117 China; 2 Jiangsu Provincial Key Laboratory of Materials Cycling and Pollution Control, School of the Environment, Nanjing Normal University, Nanjing, 210023 China; 3 Jilin Institute of Forestry Survey and Design, Changchun, 130022 China

**Keywords:** Environmental sciences, Hydrology, Engineering

## Abstract

In this paper, groundwater treatment sludge (GTS) was recycled as a magnetic adsorbent via a facile calcination process without adding any reductant. The prepared magnetic adsorbents (MAs) were characterized by scanning electron microscopy (SEM), X-ray diffractometer (XRD) and X-ray photoelectron spectroscopy (XPS), vibrating sample magnenometer (VSM) and Mössbauer spectroscopy. The results showed that GTS comprised 33.2% Fe, 1.4% Al and 6.2% Si, and exhibited a weak saturation magnetization of 0.0008 emu/g. Without NaOH, the GTS calcinated at 700 and 500 °C were well magnetized with *M*s of 20.1 and 7.1 emu/g, separately, but exhibited a low Ms of 0.43 emu/g at 300 °C. By adding NaOH powder, the *M*s of GTS apparently increased to 4.9 emu/g after calcination at 300 °C, and further to 8.5 emu/g at 500 °C. In GTS, about 96.1% Fe was involved in ferrihydrite form. The *M*s of calcinated GTS was accompanied with the phase transformation of ferrihydrite to maghemite. Si/Al oxides in GTS coordinated on the surface sites of ferrihydrite and inhibited the conjunction and phase transformation of adjacent ferrihydrite particles, but were effectively desorbed as in the presence of NaOH. Na500, preparing by calcinating GTS at 500 °C with NaOH, showed an optimal total surface sites (*H*_s_) of 0.65 mmol/g. Oxytetracycline (OTC) was used as a target for studying the adsorption characteristics of synthetic magnetic adsorbents and a high adsorption capacity of oxytetracycline of 862.1 mg/g in comparison with the other calcinated GTS, and the adsorption data was consistent with the Langmuir model. By adding 6 g/L Na-500, approximately 100% of oxytetracycline and tetracycline and nearly 40% total organic carbon were removed from real pharmaceutical wastewater. With the method, GTS can be converted in mass production to magnetic adsorbent that exhibits effective application in pharmaceutical wastewater treatment.

## Introduction

Groundwater treatment sludge is a Fe-bearing waste, mainly precipitated from the backwash wastewater in groundwater plant for tap water production^1^. In general, approximately 5t/d of sludge is generated in a groundwater plant with capacity of 50,000 m^3^/d tap water^1,2^. Such sludge is commonly disposed at dumping sites nearby, such as river, pond, deep well and sea^3,4^. Under anoxic condition, the Fe-bearing compounds in the sludge are reduced to free ferrous ion, which raise potential environmental pollution once released to water ^5^. With the legal regulation of local government, such sludge is compulsorily dewatered and then stabilized with the addition of cement reagent before safety landfill, consuming extra cost in the treatment and transport of sludge^3,6–8^.

Resource utilization of such sludge has two merits, to reduce the sludge production, and to produce new marketable products^9–11^. Accordingly, many strategies were developed to convert the sludge as products, e.g., admixture of building material^12^, iron concentrated powder, flocculants and adsorbent^2,8,10,11^. For the former three products, heavy consumption of acid/alkaline were employed to accelerate the substitute of Fe in the crystal lattice of geopolymer, and/or to dissolve the sludge as free Fe ions. Compared with the three products, recycling of the sludge as adsorbent was a promising route^2,8,10,11^. The sludge comprised Fe/Al/Si, and showed plenty of surface hydroxyl groups to adsorb cationic contaminant, e.g., heavy metals and/or cationic organics^8,11,13,14^. However, tedious precipitation and centrifugation were performed to separate the sludge form wastewater after adsorption. To make up its disadvantage, the sludge was converted to magnetic adsorbent via hydrothermal route with the addition of reductant^2,11^. For instance, in the presence of ethylene glycol^2^, the Fe oxides in the sludge was reduced to ferrous ion, followed by coprecipitating with ferric ion to form magnetic magnetite after hydrothermal treatment at 180 °C for 4 h. Other reagents, e.g., ascorbic acid^11^, pyrite^15^, and iron powder^1^, also showed similar reductive performance to ethylene glycol. Accordingly, magnetic species such as magnetite, maghemite, and jacobsite, were crystallized in the hydrothermal system. Therefore, the treated sludge showed well magnetic response and can be easily separated from wastewater in a magnetic field^10,16^.

Recently, a non-reductant route was developed to convert the sludge as magnetic adsorbent. In the conversion process, the sludge was treated with alkaline solution, where Al/Si-bearing compounds were dissolved, accelerating the thermal conversion and surface conjunction of Fe-bearing compounds in the sludge to form magnetic maghemite^2,17,18^, with hematite being final product^19–21^. However, alkaline wastewater was generated at mass production, which raises extra cost in the following wastewater treatment. The formation of maghemite also occurred at thermal condition. Such conversion was acceptable at field-scale due to the abolishment of high-pressure vessel and the elimination of alkaline wastewater. But up to date, the effect of heating temperature on the synthesis of magnetic adsorbent from groundwater treatment sludge has yet been reported^10,11,22,23^.

Herein, calcination conversion of the sludge into magnetic adsorbent was investigated. The sludge was directly calcinated at three different conditions, where the products were characterized by magnetization determination, Mössbauer experiment, and crystallization analysis. Their application in the adsorption of oxytetracycline was also investigated.

## Materials and methods

### Directly calcinating GTS to prepare magnetic adsorbents

GTS was collected at the bottom of precipitation pool of Nong-An groundwater plant (Nong-An, China). For pretreatment, GTS was dried at 105 °C for 5 h, ground to pass through 1 mm mesh, and then characterized by XRD, XRF and Mössbauer. The raw GTS was characterized by X-ray fluorescence spectrometry (XRF, XRF-1800, Shimadzu, Japan), where Fe, Si, Mn, Ca, Mg and Al were 23.2, 3.7, 0.7, 0.4, 0.06 and 7.5 wt% (Table 1), demonstrating that Si/Al were major impurities in the sludge.

**Table 1:** Composition of the sludge before and after calcination (unit: wt%).

Elements	Fe	Si	Mn	Ca	Mg	Al
Raw sludge	23.2	3.7	0.7	0.4	0.06	7.5

To prepare magnetic adsorbent, 1 g GTS powder was placed in a crucible, directly heated at 300 °C for 4 h in a muffle oven and cooled down to room temperature. Subsequently, the obtained product was washed three times with deionized water, and freeze-dried at − 80 °C overnight. The obtained sample was named as S300. Control experiments were performed by increasing the temperature from 300 to 500 and 700 °C, and the corresponded products were denoted as S500 and S700.

To enhance the magnetization of calcinated product, 0.5 g NaOH powder and 1 g GTS were homogeneously mixed in a crucible, and then treated according to the procedures described above. The treatment temperatures varied from 300 °C to 500 and 700 °C, and the related products were named as Na-300, Na-500 and Na-700, respectively.

### Estimating the adsorption performance of magnetic adsorbents

Oxytetracycline (OTC) is a typical antibiotic and commonly distributed at a high level in the effluent of pharmaceutical wastewater treatment plant^24^. Thus, OTC was targeted to estimate the adsorption performance of magnetic adsorbents in the following experiment. First, a series of stock solution of OTC was prepared with the concentration of 50–2000 mg/L, and then dispersed 0.01 g S700 in 20 mL stock solution followed by stirring at 140 rpm overnight. Second, the S700 was separated after adsorption by a magnet, freeze-dried and further characterized. Third, the supernatant was collected and the residual OTC was determined by high-performance liquid chromatograph (HPLC). The OTC concentration was determined using a high-performance liquid chromatographer (Waters-2695, Waters Alliance, USA) with a C18 column (ODS–C18, 46 mm × 255 mm, Waters Alliance, USA), followed by UV detection at 360 nm. A mixture of 0.01 M oxalic acid–acetonitrile (80:20, v/v) was used as mobile phase with a flow rate of 1 mL/min. The retention time was 5.25 min, and the detection limit was 0.01 mg/L.

In parallel, other magnetic adsorbents such as S500, Na300, Na500 and Na700 were also tested with the abovementioned procedures.

The total surface sites (*H*_s_) of prepared adsorbents were also determined by potentiometric titration according to the method of Zhu et al.^23^. To perform the titration experiment, 0.2 g S700 was mixed with 50 mL 0.01 M NaNO_3_ solution in a beaker, bubbled with nitrogen gas constantly, and then titrated the solution to pH 3 with 0.2 M nitric acid, followed by titrating to pH 11 with 0.2 M NaOH. To calculate the *H*_s_ of S700, Gran plot according to the Eqs. (1) and (2) was performed to calculate the Gran function value, and the *H*_s_ of S700 was finally calculated by the Eq. (3). Same method was used to calculate the *H*_s_ of the other adsorbents.

1 $$G=\left({V}_{0}+{V}_{1}+{V}_{2}\right)\times {10}^{-pH}, \mathrm{at \; acidic \;condition},$$

2 $$G=\left({V}_{0}+{V}_{1}+{V}_{2}\right)\times {10}^{-13.8+pH}, \mathrm{at \;alkaline \;condition},$$

3 $${H}_{s}=\frac{({V}_{e2}-{V}_{e1}{)}_{MA}-({V}_{e2}-{V}_{e1}{)}_{R}}{m}\times {C}_\text{NaOH},$$

In the equations, *V*_0_, *V*_1_, and *V*_2_ are the volumes (mL) of the NaNO_3_, the consumed nitric acid and NaOH solution; *V*_e2_ and *V*_e1_ are the added NaOH volume (mL) for neutralizing the total H^+^ and the free H^+^ in the titration system; m is the adsorbent weight (g).

### Application of the magnetic adsorbents in pharmaceutical wastewater treatment

Pharmaceutical wastewater was acquired from the influent of wastewater treatment station (Lihua pharmaceutical co., China). Na500 had the highest *H*_s_ and the optimal OTC adsorption capacity, and was used in wastewater treatment. To treat wastewater, 0.1 g Na500 was added in 100 mL wastewater and stirred at 140 rpm overnight. The used Na500 was collected and then determined the concentrations of OTC, tetracycline (TC) and total organic carbon (TOC) in the treated wastewater. To optimize the treatment efficiency, the dosage of Na500 varied from 0.1 to 10 g in the control experiments according to the above steps.

## Results and discussion

### Transformation of Fe oxides in GTS to magnetic species

**Figure 1:**
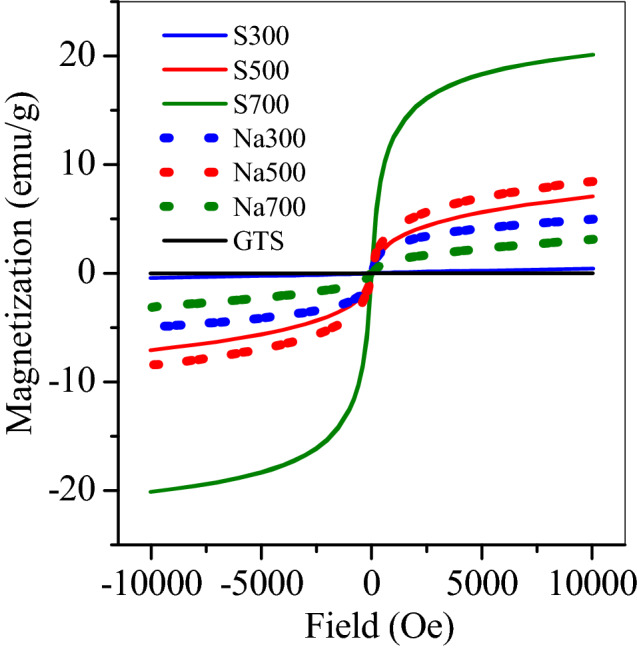
Hysteresis loops of GTS and the prepared adsorbents.

GTS was Fe-bearing precipitate from groundwater treatment plant, and showed a weak saturation magnetization of 0.0008 emu/g. After calcination, the *M*_s_ of prepared adsorbents apparently increased to 0.43, 7.1 and 20.1 emu/g with temperature increasing from a range of 300 to 500 and 700 °C (Fig. 1 S300, S500 and S700), separately. This indicated that the magnetic species was generated in GTS after directly calcination and its generation accelerated at high temperature. By adding NaOH, Na300, prepared by calcinating at 300 °C, showed a high *M*_s_ of 4.9 emu/g, nearly 10 times of that of S300. This demonstrated that NaOH also accelerated the formation of magnetic species in GTS. In the presence of NaOH, the product *M*_s_ also increased further to 8.5 emu/g at 500 °C, but dropped to 3.1 emu/g at 700 °C, which was probably related to the change of magnetic species in GTS.

**Figure 2:**
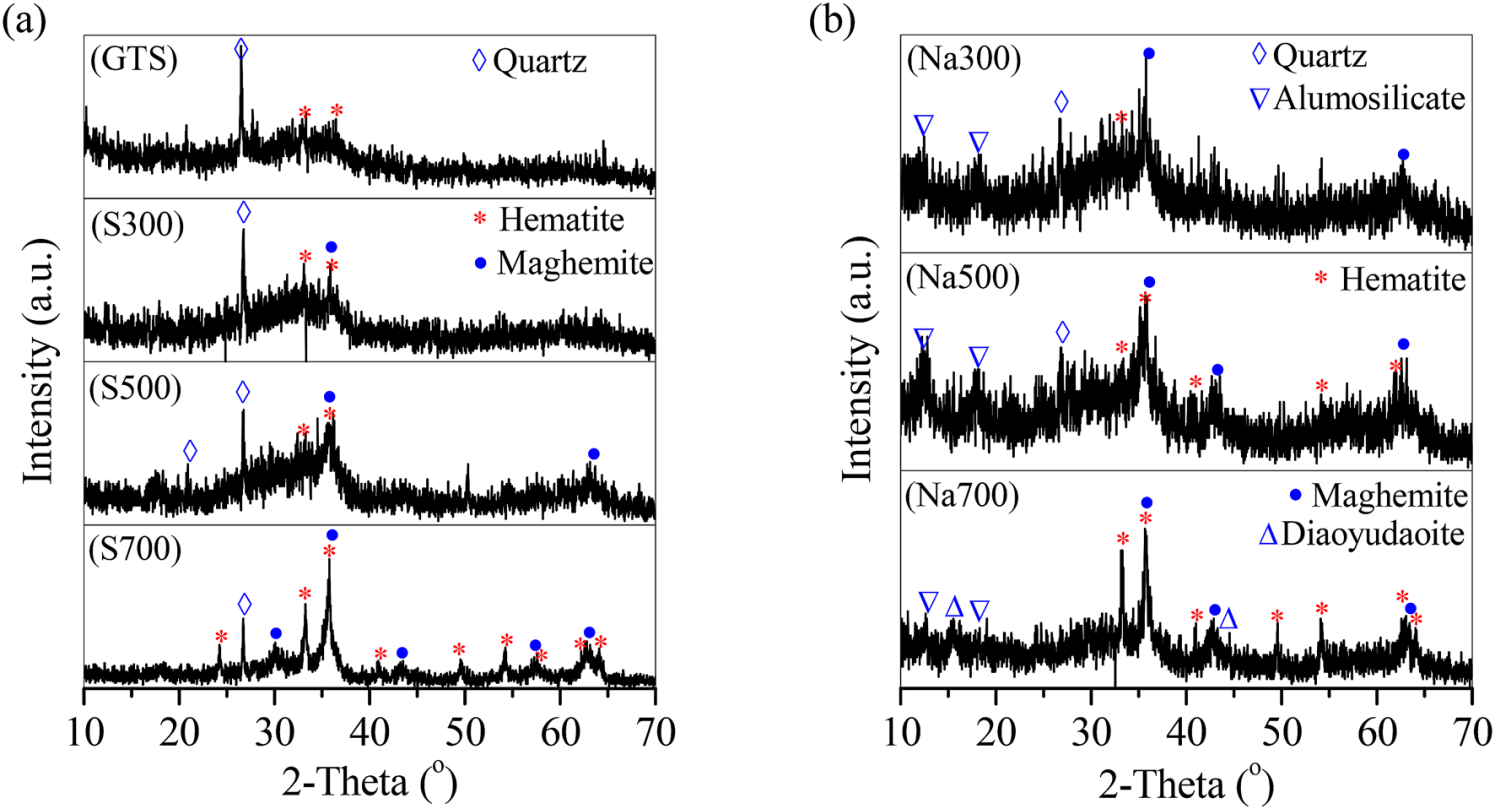
XRD curves of the GTS and prepared adsorbents.

**Figure 3:**
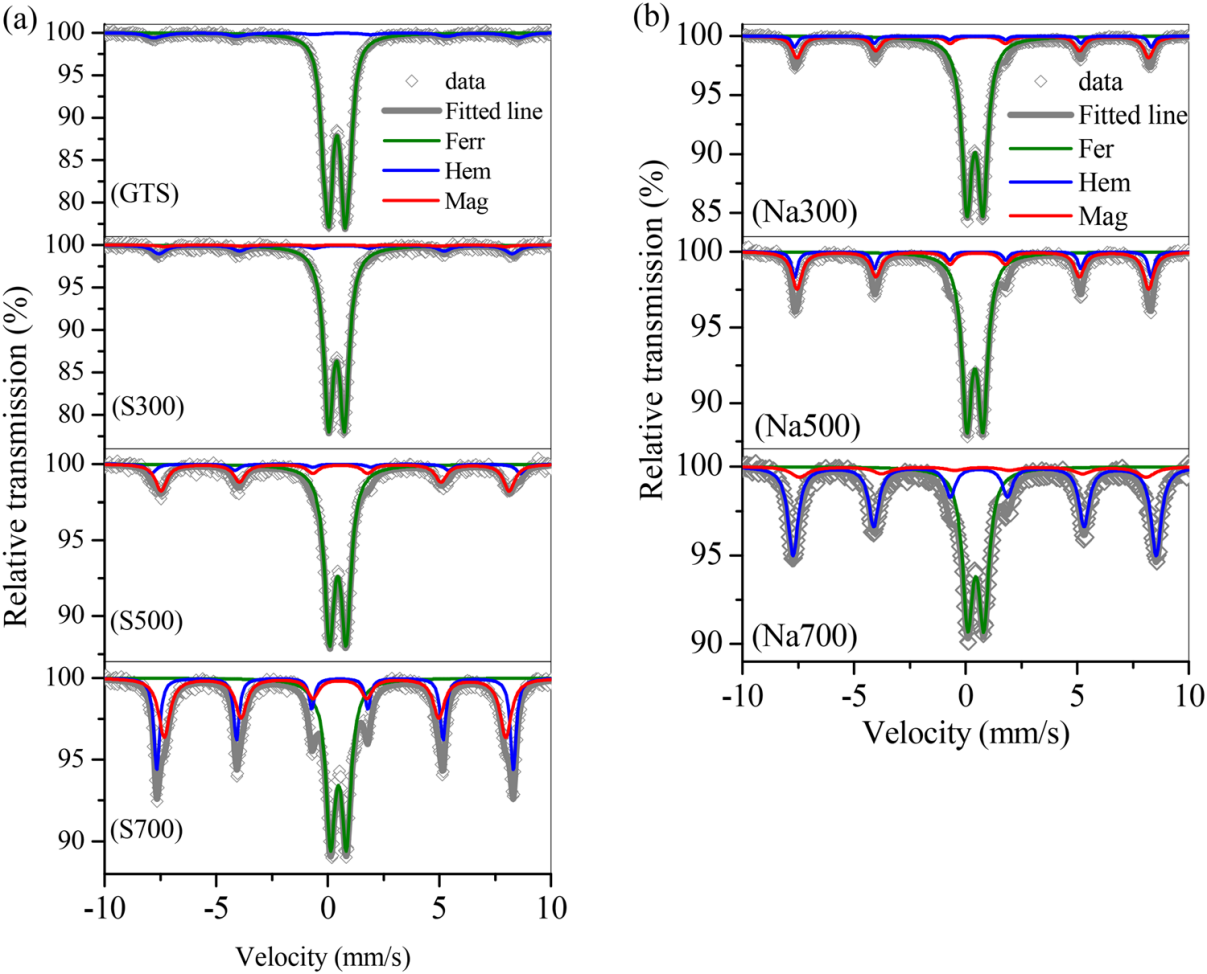
Mössbauer spectra of (**a**) GTS, S300, S500, S700, and (b) Na300, Na500 and Na700.

The magnetic species in the magnetic adsorbents were characterized by XRD and Mössbauer, as shown in Figs. 2 and 3. In GTS, Fe oxides can be categorized to hematite (Fig. 2a) and ferrihydrite (Fig. 3 GTS). No magnetic species were observed in GTS, corresponding to the weak *M*_s_ of GTS. However, the relative percentage of ferrihydrite in Fe oxides was 96.1% (Table 2), revealing that ferrihydrite was predominant. After calcination, the calcinated products showed that a new peak of Fe oxides at 2θ = 35.7° (Fig. 2a) belonged to maghemite. Maghemite was a typical magnetic species, and its relative percentage in Fe oxides in calcinated product was accompanied by the *M*_s_ change. As shown in Fig. 3, with the temperature increasing from 300 to 500 and 700 °C range, the relative percentage of maghemite gradually increased from 1.22 to 18.4% and 32.1%, which showed a similar increased to the *M*_s_. Moreover, the hematite percentage also elevated from 8.5 to 11.5% and 22.6%, whilst the ferrihydrite percentage decreased from 90.3 to 70.2% and 45.4% (Fig. 3 and Table 2), demonstrating that ferrihydrite was converted to maghemite and hematite, and its conversion accelerated with the temperature increasing from 300 to 700 °C in calcination process.

**Table 2:** Relative content of minerals in Fe oxides of GTS and the treated products.

	Minerals	Relative percentage (%)
GTS	Ferrihydrite	96.10
	Hematite	3.90
S300	Ferrihydrite	90.26
	Hematite	8.52
	Maghemite	1.22
S500	Ferrihydrite	70.18
	Hematite	11.45
	Maghemite	18.37
S700	Ferrihydrite	45.35
	Hematite	22.60
	Maghemite	32.05
Na300	Ferrihydrite	74.75
	Hematite	10.65
	Maghemite	14.60
Na500	Ferrihydrite	58.74
	Hematite	17.50
	Maghemite	23.76
Na700	Ferrihydrite	37.29
	Hematite	52.60
	Maghemite	10.11

By adding NaOH, the obtained products also showed a clear peak of maghemite (Fig. 2b). The maghemite percentage was 14.6% at 300 °C, peaked 23.8% at 500 °C, but dropped to 10.1% at 700 °C, similar to the change of *M*_s_ (Fig. 1). Moreover, in the range of 500–700 °C, the ferrihydrite percentage decreased steadily from 58.7 to 37.3%, but the hematite percentage considerably increased from 17.5 to 57.6% (Fig. 3 and Table 2), suggesting that the conversion of ferrihydrite continued at 700 °C, to generate final product hematite, with maghemite as the intermediate.

Si/Al oxides were the major impurities in GTS, but only well crystallized quartz was observed in GTS curve (Fig. 2a). The quartz peak kept unchanged in the calcination process without adding NaOH (Fig. 2a), but gradually disappeared in the presence of NaOH (Fig. 2b). Instead, new peaks at 2θ = 12.3° and 18.2° affiliated to alumosilicate, were observed in the curves of Na300 and Na500. With the temperature increasing to 700 °C, the product Na700 showed the two peaks of alumosilicate and a new peak of diaoyudaoite at 15.5° (Fig. 2b). This indicated the transformation of Si/Al oxides in GTS (e.g. quartz) to alumosilicate at 300–500 °C and further to diaoyudaoite at 700 °C with the addition of NaOH.

**Figure 4:**
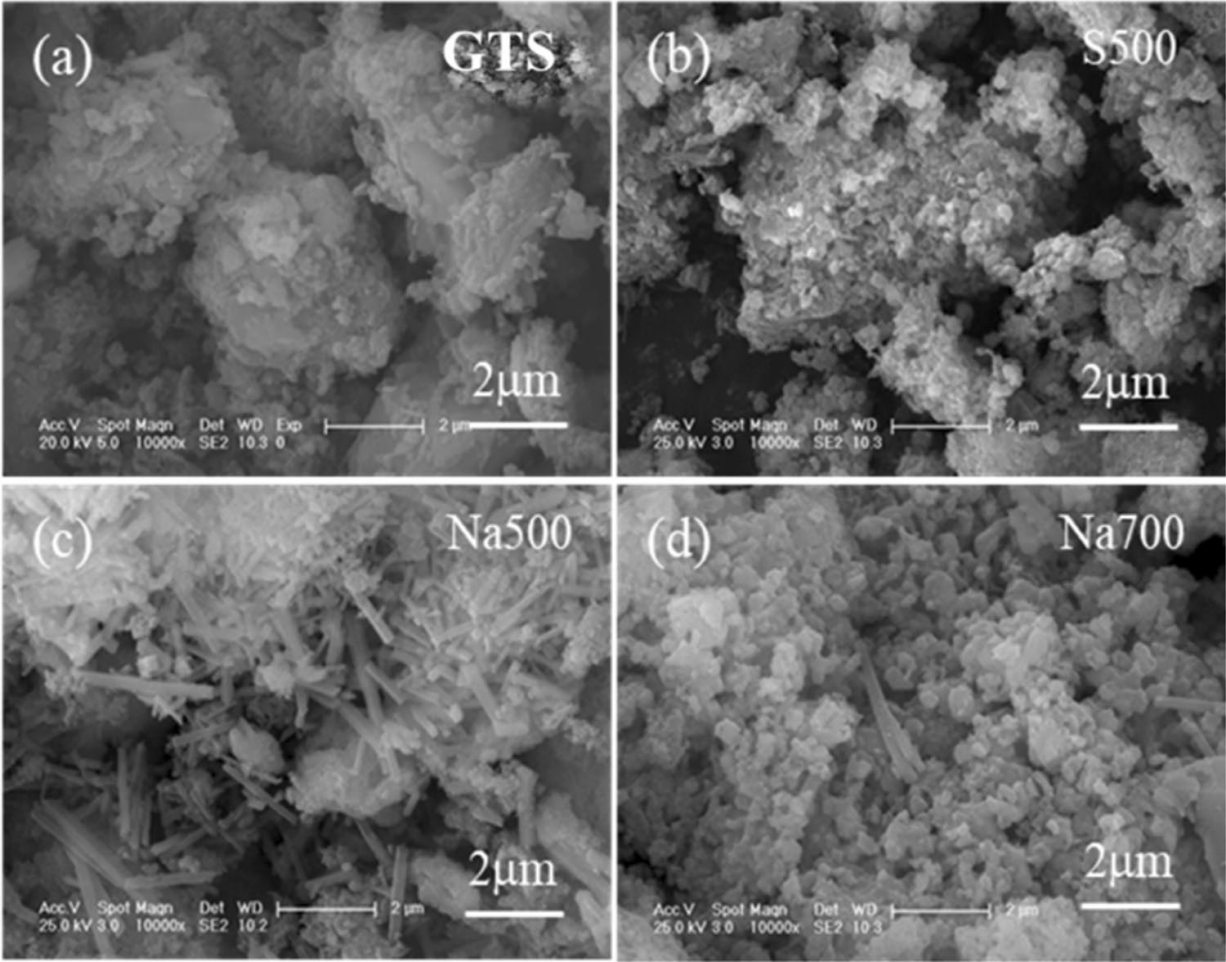
SEM pictures of the GTS and prepared adsorbents.

GTS was irregular aggregates (Fig. 4a), and did not change apparently after directly calcination at 500 °C (Fig. 4b). By adding NaOH, nanorod with length of 0.5–5 μm aside the irregular aggregates appeared in Na500 (Fig. 4c), in accordance with the formation of alumosilicate (Fig. 2b). But as the temperature increased to 700 °C, Na700 showed that nanoparticles aggregated along with few nanorod particles (Fig. 4d), which was assigned to the conversion of alumosilicate to diaoyudaoite (Fig. 2b).

GTS is precipitated from the Fe-bearing groundwater after treatment by gas aeration and coagulation with polyaluminium^26,27^. The ferrous Fe in groundwater is oxidized by dissolved oxygen in gas aeration process to generate ferric Fe^28^, and further hydrolyzes in the form of Fe oxyhydroxide aggregate. Such aggregate is suspended as fine particles in water and had plenty of surface hydroxyl groups^29,30^, and can be easily coagulated with the hydrolyzed PAC to precipitate from water. Thus, the surface sites of Fe-bearing aggregate (mainly in ferrihydrite) was covered by Al/Si compounds. After calcination, the rearrangement of structural Fe and O atom in ferrihydrite occurred, to release excess water molecules and to form new Fe–O–Fe bond, which led to form maghemite with hematite as final product. Under high temperature, the release of excess water molecules accelerated, which promoted the combination of Fe–O–Fe bond and the following phase transformation of ferrihydrite to maghemite and hematite. Therefore, S700 showed the optimal *M*_s_.

When NaOH was introduced in the calcination system, Al/Si compounds on ferrihydrite surface were easily reacted with NaOH under high temperature to generate alumosilicate mixture, which recovered the free hydroxyl groups of ferrihydrite. In the following step, the conjunction of two free hydroxyl groups on the adjacent ferrihydrite surface took place, to release one water molecule and form Fe–O–Fe bond. As the conjunction reaction continued, the transformation of ferrihydrite to maghemite and hematite achieved. Wen et al. investigated the calcination of pure ferrihydrite, and found a rapid transformation of pure ferrihydrite to hematite with maghemite as intermediate^31,32^. At 700 °C, most of Al/Si compounds were involved in the formation of alumosilicate and diaoyudaoite in the presence of NaOH, and thus the conversion of maghemite to hematite also accelerated, resulting in a low *M*_s_ of the product Na700.

### Adsorption capacity of the prepared adsorbents

**Figure 5:**
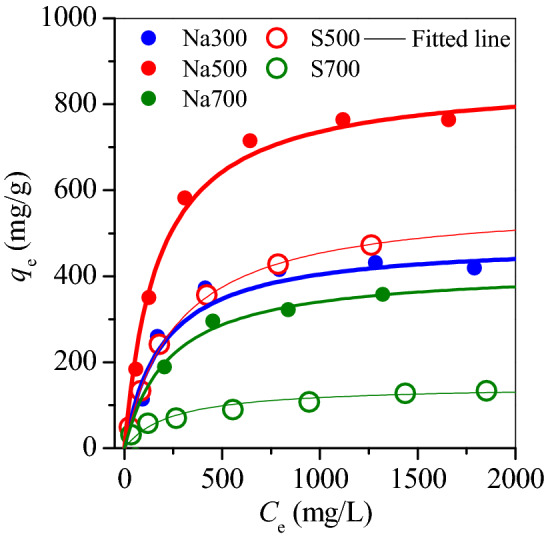
Linear fitting of oxytetracycline adsorption on the prepared magnetic adsorbents.

**Figure 6:**
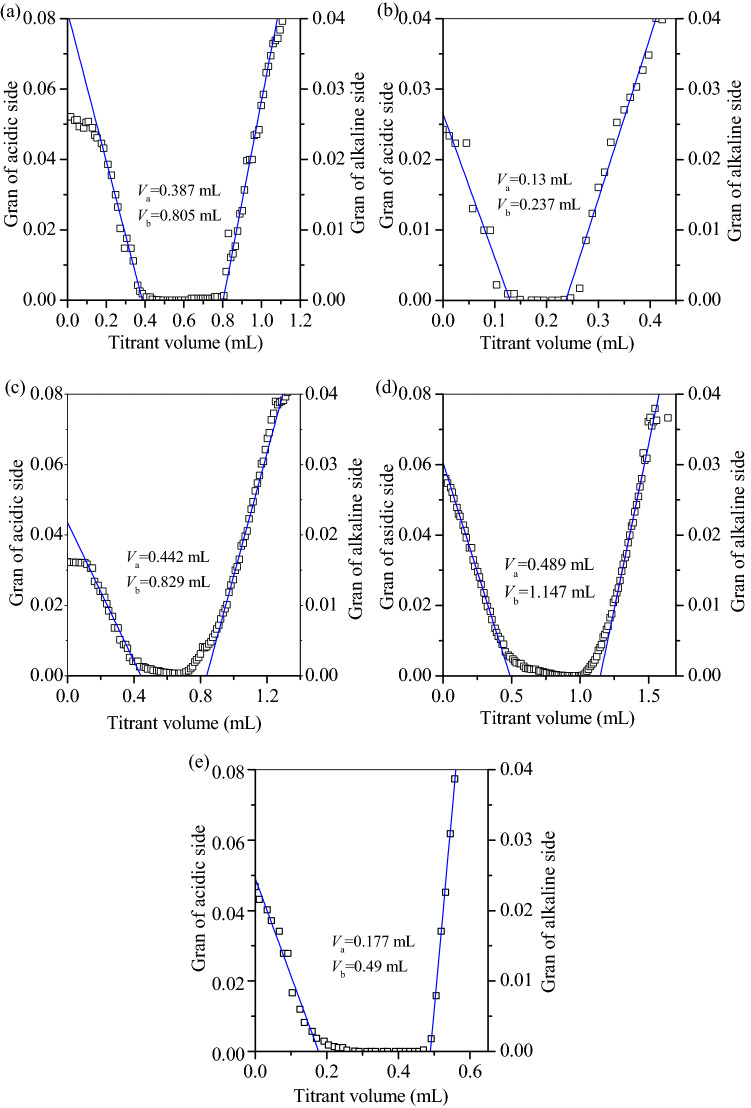
Gran plots of (**a**) S500, (**b**) S700, (**c**) Na300, (**d**) Na500 and (**e**) Na700.

Oxytetracycline is a common antibiotic in pharmaceutical wastewater, and was targeted to determine the adsorption capacity of prepared adsorbents as shown in Fig. 5. The adsorption data fits well with Langmuir isotherm models (Fig. 5) with correlation coefficient (R^2^) > 0.9, demonstrating that the prepared adsorbents had an energy-balanced surface. The maximum adsorption capacity (*q*_m_) of OTC was in the following order: Na500 > S500 > Na300 > N700 > S700 (Table 3), similar to the change of *H*_s_ (Fig. 6), demonstrating that the adsorbent surface sites played a key role in the adsorption of OTC.

**Table 3:** Parameters of the prepared magnetic adsorbents.

	Parameters	S500	S700	Na300	Na500	Na700
Langmuir isotherm	*R*^2^	0.999	0.982	0.985	0.996	0.994
	*q*_max_	574.7	146	483.1	862.1	418.4
	*K*_L_	0.0037	0.0042	0.0051	0.0058	0.0044
Gran plot	*H*_s_	0.41	0.1	0.38	0.65	0.3

The *q*_m_ and *H*_s_ of S500 was higher than S700, but lower than Na500. This indicated that the direct calcination agglomerated GTS particles and decreased the total number of surface sites of product. By adding NaOH, the surface Al/Si compounds were converted to alumosilicate nanorod (Fig. 4c), which was important for the total number of surface sites of Na500. With the temperature increased from 500 to 700 °C, alumosilicate nanorod was further converted to aggregated diaoyudaoite. With the aggregation, *H*_s_ of Na700 apparently decreased, in agreement with the decrease of surface functional sites. Accordingly, Na700 showed a low value of *q*_m_ in comparison with Na500.

### The effect of solution pH

**Figure 7:**
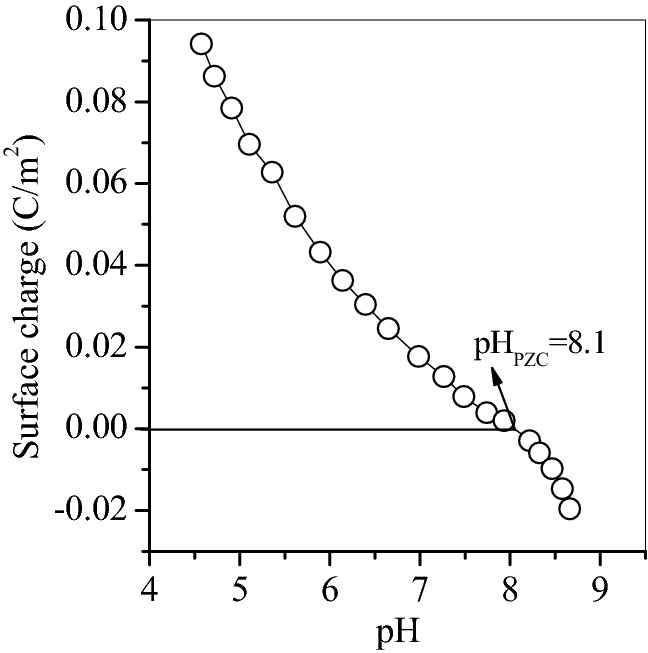
pH_PZC_ of magnetic adsorbent Na500.

The prepared adsorbent Na500 exhibited well magnetic response and high adsorption capacity of oxytetracycline, and its pH_PZC_ was also determined and the result was shown in Fig. 7.

The pH_ZPC_ of Na500 is 8.1 after calculating with the method of Kosmulski et al.^33^ as shown in Fig. 7. After adsorption, the solution was in the pH range of 6–7.8, and accordingly the added Na500 showed positively charged surface. However, oxytetracycline was in the zwitter-ion form, and easily diffused to the surface of Na500 without any electrostatic repulsion. Thus, the complexation reaction between oxytetracycline and surface hydroxyl groups of oxytetracycline occurred.

### Adsorption mechanism of oxytetracycline on the prepared adsorbents

**Figure 8:**
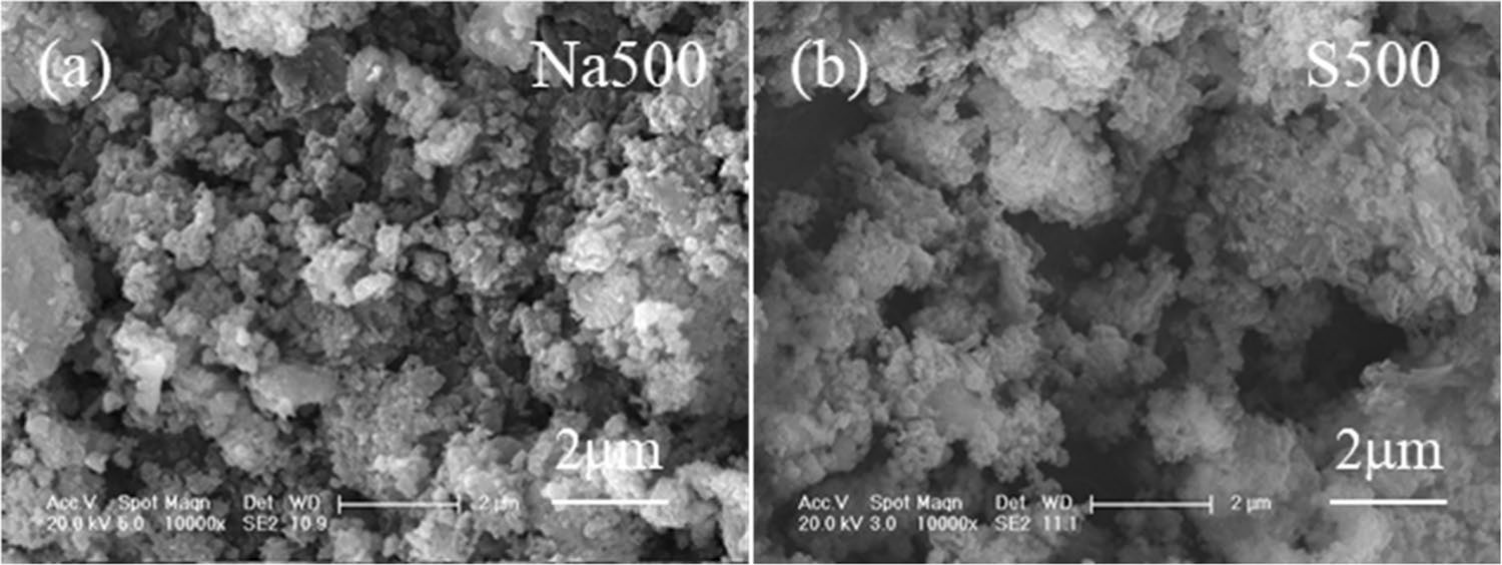
SEM pictures of (**a**) Na500 and (**b**) S500 after OTC adsorption.

**Figure 9:**
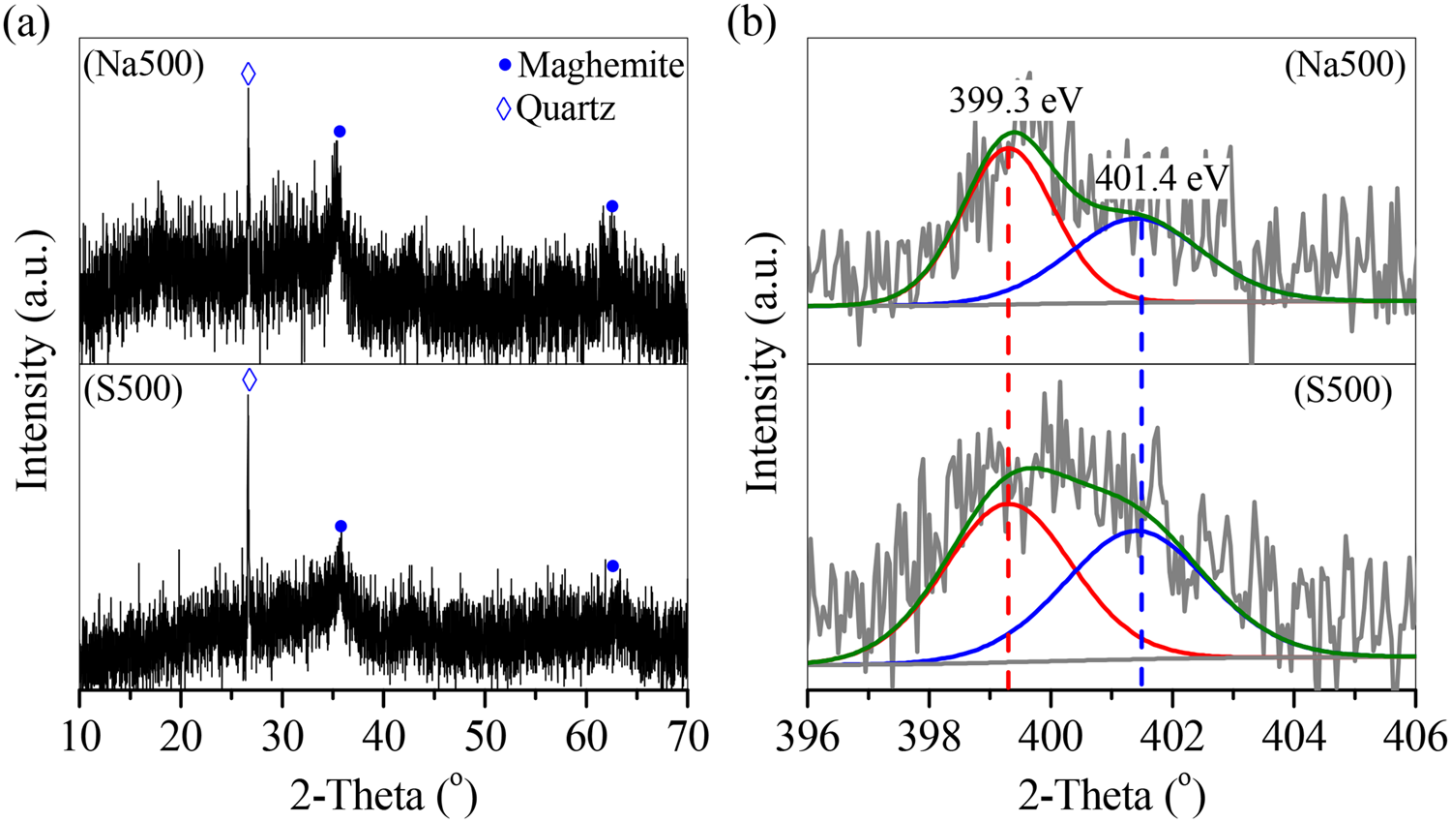
(**a**) XRD and (**b**) N1s XPS curves of Na500 and S500 after adsorption.

To analyze the adsorption mechanism of OTC, Na500 and S500 after OTC adsorption was characterized by SEM, XRD and XPS. Na500 showed that the alumosilicate nanorod (Fig. 8a) and its XRD peaks (Fig. 9a) disappeared, due to the hydrolysis of alumosilicate nanorod. Only peaks of quartz and maghemite were observed (Fig. 9a), whilst hematite peaks may be covered by the hydrolyzed alumosilicate. However, the morphology, XRD and XPS peaks of S500 kept almost unchanged (Figs. 8b, 9a and Fig. S1), suggesting that S500 was stable during OTC adsorption. The XPS spectra of Na500 and S500 showed that two peaks at the binding energy of 399.3 eV and 401.4 eV (Fig. 9b), which belonged to the N atom in –NH_3_^+^ and –NH– of OTC, respectively.

**Figure 10:**
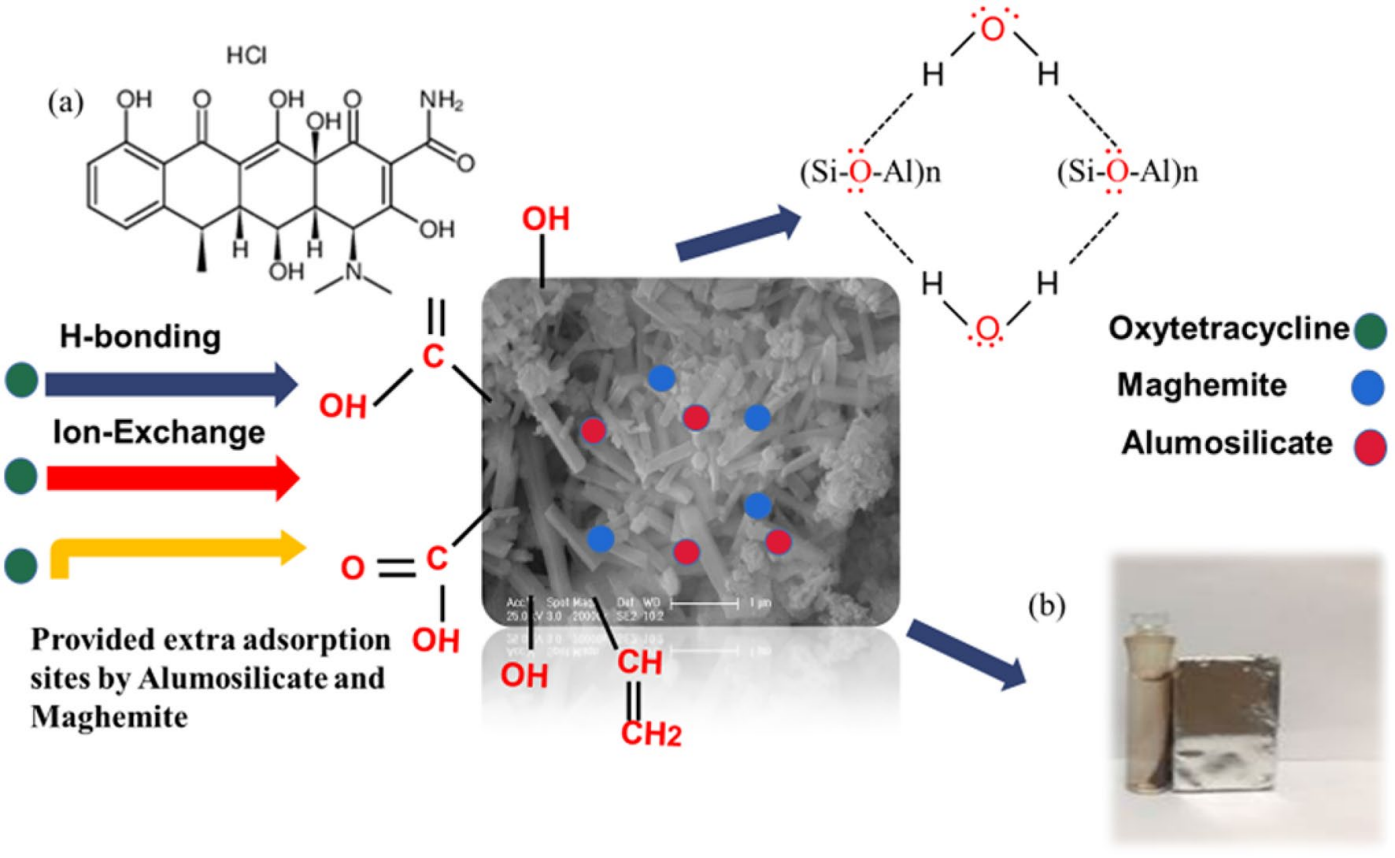
The adsorption mechanism of OTC on Na500, (**a**) OTC structure, (**b**) magnetic separation of adsorbents after use.

OTC is a zwitterionic antibiotic, which is cation at pH < 3.3, zwitterion in the pH range of 3.3–8, and anion at pH > 7.7. After adsorption, the pH of residual solution was ranged from 6 to 7.8. Therefore, OTC was in the zwitterionic form and easily diffused to the surface of adsorbents (e.g. Na500 and S500). Subsequently, the coordination reaction between the -NH_2_ group on the side chain of OTC and the surface hydroxyl groups of adsorbents occurred, resulting in the adsorption of OTC (Fig. 10). The product S500 was also a mixture of Fe, Al and Si, and thus exhibited three hydroxyl groups, ≡Fe-OH, ≡Al-OH and ≡Si-OH, for OTC adsorption. Na500 showed similar hydroxyl groups to S500, but alumosilicate nanorod in Na500 was metastable and spontaneously to generate Al/Si-bearing oxyhydroxides. Such Al/Si-bearing oxyhydroxides had plenty of hydroxyl groups in comparison with solid particles, to coordinate more OTC molecules (Fig. 10).

### Environmental application

The prepared adsorbent had plenty of surface functional groups, e.g., ≡Fe-OH, ≡Al-OH and ≡Si-OH, and showed superior efficiency in the removal of tetracycline and oxytetracycline from pharmaceutical wastewater. Such groups were also effective in the adsorption of cationic organics and heavy metals, e.g., Zn, Cu, and Ni. Such magnetic adsorbent can be easily separated from wastewater in the help of magnetic field (Fig. 10b), which employed a facile route to rapid recycle used adsorbent.

With the method, GTS was facilely converted to magnetic adsorbent without generating any secondary waste, which employed a green route to recycle other Fe-rich sludges, e.g., cold-rolling sludge and Fenton sludge. Only heavy energy was consumed in the calcination process, and thereby such method was acceptable in the power station and/or heating plant with the adequate of waste heat. Although the method had advantage to reduce the sludge production and to produce new magnetic adsorbent, two important issues should be focused on in the future research. One is to regenerate the used adsorbent, for the enrichment of adsorbed contaminants; The other is to graft functional groups on the magnetic adsorbent, so that the prepared adsorbent has selective adsorption performance in the wastewater treatment.

**Figure 11:**
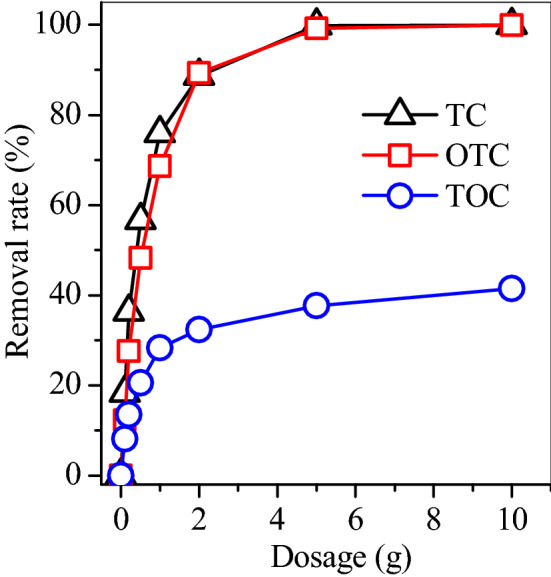
Dosage of Na500 on the treatment of pharmaceutical wastewater.

Na500 had optimal *q*_m_ of OTC and *H*_s_ among the five magnetic adsorbents, and thus was employed to treat real pharmaceutical wastewater. The pharmaceutical wastewater contained 21 mg/L OTC, 6.2 mg/L tetracycline, and 288.6 mg/L total organic carbon (TOC). As the Na500 dosage increased to 6 g, nearly 100% OTC and TC, and about 40% TOC was removed (Fig. 11). This demonstrated that Na500 was efficient to remove TC, OTC and other cationic organics in pharmaceutical wastewater.

### Comparative study of OTC

Table 4 compares the adsorption capacity of other adsorbents found in the literature with OTC. The results show that the adsorption performance of synthesized magnetic adsorbent (Na500) is comparable to other adsorbents, so it is suitable for removing OTC from wastewater. Considering the variations within the physical properties of the material and also the experimental conditions, it's quite tough to form a good assessment victimisation the on top of knowledge, though our knowledge still offer helpful info, at least at a level of relevance.

**Table 4:** Comparison of adsorption capability of OTC on Na500 with other adsorbents.

Adsorbent	Concentration (mg/L)	*q*_m_ (mg/g)	Refs.
Na500	2000	862.1	This work
Commercial activated carbon	100–1000	413.2	^25^
Activated carbon fibre	100	312.5	^34^
Graphene oxide	400	212.3	^35^
Multiwalled carbon nanotubes	1000	190.2	^36^
Graphene oxide functionalized magnetic particles	50	45.0	^37^
Illite/kaolinite	30–60	8.85/4.75	^38^
Chitosan-coated fly ash composite	10–300	291.3	^39^
Activated sludge	500	72	^40^

Finally, due to the relatively low cost of starting materials and routes used, the resulting synthesized magnetic adsorbent (Na500) may be cost-effective sorbents for over-the-counter removal of aqueous solutions. The *q*_m_ of OTC on Na500 was 852.1 mg/g, apparently higher than adsorbents.

### Estimation cost of adsorbent production

Compared with the reported hydrothermal route, calcination heavily consumed extra energy, and its operation cost was calculated as shown in Table 5. The treatment of 1 ton sludge consumed 0.5 ton of sodium hydroxide and 30 kWh power for heating, which amounted to US$200, but US$ 66.1 could be saved from disposing the sludge to landfill^8^, which could be deducted from the treatment cost of sludge. The produced adsorbent showed good adsorption capacity of oxytetracycline in comparison with commercial adsorbents, e.g., graphene oxide functionalized magnetic particles, and graphene oxide, and thus can be served as a marketable adsorbent in the wastewater treatment. Subsequently, this route cancelled the hydrothermal vessel and the drying of adsorbent, and did not generate secondary alkaline wastewater, in comparison with hydrothermal route and was promising in the field-scale application.

**Table 5:** Operation cost of 1 ton magnetic adsorbent for OTC treatment.

Reagent and processing	Prize	Usage per ton	Total
Sodium hydroxide	262 US$/t	0.5t	131
Power	0.23 US$/kW h	30 kW h (total 10 h)	69
Total			200US$
Iron mud disposal	1800 US$/t	8.2 kg	14.76
Poly(acrylamide)	0.23 US$/kW h	18 kW h (about 1 h)	4.14
Shipping	0.5 US$/t km	58 km	29
Landfill	18.2 US$/t		18.2
Total			66.1

## Conclusion

Groundwater treatment sludge is a solid waste comprising ferrihydrite, hematite, quartz and other impurities. With calcination treatment, the conversion of ferrihydrite to hematite occurred, with maghemite being intermediate, and accordingly the obtained product exhibited well magnetic response. Such conversion accelerated in the presence of NaOH, which initiated in the following steps: (1) the recrystallization of Si/Al oxides on Fe oxides surface, (2) the regeneration of free hydroxyl groups of Fe-bearing microcrystal, (3) the conjunction reaction of adjacent hydroxyl groups as Fe–O–Fe bond, (4) the continued reaction of Fe–O–Fe bond to form maghemite and hematite. The product Na500, prepared by adding NaOH at the calcination temperature of 500 °C, showed a desirable magnetization of 8.5 emu/g and the optimal adsorption capacity of oxytetracycline (862.1 mg/g). In the absence of NaOH, the product S500 showed a magnetization of 7.1 emu/g and adsorption capacity of 574.7 mg/g oxytetracycline, lower than these of Na500. The adsorbent Na500 showed effective removal efficiencies in the removal of tetracycline and oxytetracycline from pharmaceutical wastewater.

### Novelty

In the reported literatures, hydrothermal route was predominant, and employed to recycle the sludge as magnetic adsorbent, in which alkaline solution was used and residual as wastewater. Especially, in the hydrothermal system, the involvement of costly reductant in the hydrothermal system apparently increased the total cost of magnetic adsorbent. Moreover, the application of hydrothermal vessel in field-scale is difficult.

Compared with the hydrothermal route, calcination was easily operated at field-scale, and effectively performed without any reductant. Even though extra energy was consumed, the calcination process eliminated the drying of magnetic adsorbent without generating any secondary wastewater, and thereby showed superior efficiency in the recycling of sludge.

## Supplementary Information

Supplementary Information.

## References

[1] Ngatenah, S., Kutty, S. & Isa M. Optimization of heavy metal removal from aqueous solution using groundwater treatment plant sludge (GWTPS). In *Proceedings of the International Conference on Environment; 2010 Dec 13–15; Penang, Malaysia* 1–9 (2010).

[2] Zhu S (2015). A novel conversion of the groundwater treatment sludge to magnetic particles for the adsorption of methylene blue. J. Hazard. Mater..

[3] Liu X, Zhang N (2011). Utilization of red mud in cement production: A review. Waste Manag. Res..

[4] Sotero-Santos RB, Rocha O, Povinelli J (2005). Evaluation of water treatment sludges toxicity using the Daphnia bioassay. Water Res..

[5] Mayes WM (2016). Advances in understanding environmental risks of red mud after the Ajka spill, Hungary. J. Sustain. Metall..

[6] Osman, S.B.S. & Iqbal. F. Possible stabilization of sludge from groundwater treatment plant using electrokinetic method. In *Applied Mechanics and Materials*. (2014) Trans Tech Publ.

[7] Zhang L (2019). Effects of Al^3+^ on pollutant removal and extracellular polymeric substances (EPS) under anaerobic, anoxic and oxic conditions. Front. Environ. Sci. Eng..

[8] Zhu S (2018). Hydrothermal synthesis of a magnetic adsorbent from wasted iron mud for effective removal of heavy metals from smelting wastewater. Environ. Sci. Pollut. Res..

[9] Qu Z (2020). Upcycling of groundwater treatment sludge to magnetic Fe/Mn-bearing nanorod for chromate adsorption from wastewater treatment. PLoS ONE.

[10] Qu Z (2019). Green synthesis of magnetic adsorbent using groundwater treatment sludge for tetracycline adsorption. Engineering.

[11] Zhu S (2019). Valorization of manganese-containing groundwater treatment sludge by preparing magnetic adsorbent for Cu (II) adsorption. J. Environ. Manag..

[12] Hu Y (2019). Role of Fe species in geopolymer synthesized from alkali-thermal pretreated Fe-rich Bayer red mud. Constr. Build. Mater..

[13] Geng Z (2017). Comparing polyethersulfone and polyurethane-immobilized cells of comamonas testosteroni QYY in treatment of an accidental dye wastewater. Chem. Res. Chin. Univ..

[14] Zhang H (2019). Fate of NaClO and membrane foulants during in-situ cleaning of membrane bioreactors: Combined effect on thermodynamic properties of sludge. Biochem. Eng. J..

[15] Liu Y (2014). Recycling of iron from red mud by magnetic separation after co-roasting with pyrite. Thermochim. Acta.

[16] Bian R (2019). Resource recovery of wastewater treatment sludge: Synthesis of a magnetic cancrinite adsorbent. RSC Adv..

[17] Li X-B (2015). Conversion of ferric oxide to magnetite by hydrothermal reduction in Bayer digestion process. Trans. Nonferrous Metals Soc. China.

[18] Mohammed M, Shitu A, Ibrahim A (2014). Removal of methylene blue using low cost adsorbent: A review. Res. J. Chem. Sci. ISSN.

[19] Ghaedi M (2015). Modeling of competitive ultrasonic assisted removal of the dyes–methylene blue and safranin-O using Fe_3_O_4_ nanoparticles. Chem. Eng. J..

[20] Rajendran S (2016). Ce^3+^-ion-induced visible-light photocatalytic degradation and electrochemical activity of ZnO/CeO_2_ nanocomposite. Sci. Rep..

[21] Saravanan R (2013). Enhanced photocatalytic activity of ZnO/CuO nanocomposite for the degradation of textile dye on visible light illumination. Mater. Sci. Eng. C.

[22] Yang L (2015). Material prepared from drinking waterworks sludge as adsorbent for ammonium removal from wastewater. Appl. Surf. Sci..

[23] Zhu S (2020). Green synthesis of magnetic sodalite sphere by using groundwater treatment sludge for tetracycline adsorption. J. Clean. Prod..

[24] Bhaumik M (2011). Enhanced removal of Cr (VI) from aqueous solution using polypyrrole/Fe_3_O_4_ magnetic nanocomposite. J. Hazard. Mater..

[25] Rivera-Utrilla J (2013). Tetracycline removal from water by adsorption/bioadsorption on activated carbons and sludge-derived adsorbents. J. Environ. Manag..

[26] Setshedi KZ (2013). Exfoliated polypyrrole-organically modified montmorillonite clay nanocomposite as a potential adsorbent for Cr(VI) removal. Chem. Eng. J..

[27] Zheng C (2018). Synthesis of novel modified magnetic chitosan particles and their adsorption performance toward Cr(VI). Bioresour. Technol..

[28] Du Y (2018). Adsorption and photoreduction of Cr(VI) via diatomite modified by Nb_2_O_5_ nanorods. Particuology.

[29] Duan S (2017). Synthesis of magnetic biochar from iron sludge for the enhancement of Cr(VI) removal from solution. J. Taiwan Inst. Chem. Eng..

[30] Xiao Y, Liang H, Wang Z (2013). MnFe_2_O_4_/chitosan nanocomposites as a recyclable adsorbent for the removal of hexavalent chromium. Mater. Res. Bull..

[31] Hu J, Lo IM, Chen G (2005). Fast removal and recovery of Cr(VI) using surface-modified jacobsite (MnFe_2_O_4_) nanoparticles. Langmuir.

[32] Wen Y (2011). Adsorption of Cr(VI) from aqueous solutions using chitosan-coated fly ash composite as biosorbent. Chem. Eng. J..

[33] Kosmulski M (2009). Compilation of PZC and IEP of sparingly soluble metal oxides and hydroxides from literature. Adv. Coll. Interface. Sci..

[34] Huang L (2013). Characterization of activated carbon fiber by microwave heating and the adsorption of tetracycline antibiotics. Sep. Sci. Technol..

[35] Gao Y (2012). Adsorption and removal of tetracycline antibiotics from aqueous solution by graphene oxide. J. Colloid Interface Sci..

[36] Oleszczuk P, Pan B, Xing B (2009). Adsorption and desorption of oxytetracycline and carbamazepine by multiwalled carbon nanotubes. Environ. Sci. Technol..

[37] Lin Y, Xu S, Li J (2013). Fast and highly efficient tetracyclines removal from environmental waters by graphene oxide functionalized magnetic particles. Chem. Eng. J..

[38] Bansal, O. Sorption of tetracycline, oxytetracycline, and chlortetracycline in illite and kaolinite suspensions. International Scholarly Research Notices, 2013 (2013).

[39] Harja M, Ciobanu G (2018). Studies on adsorption of oxytetracycline from aqueous solutions onto hydroxyapatite. Sci. Total Environ..

[40] Prado N, Ochoa J, Amrane A (2009). Biodegradation and biosorption of tetracycline and tylosin antibiotics in activated sludge system. Process Biochem..

