# Supplementary material for: A facile calcination conversion of groundwater treatment sludge (GTS) as magnetic adsorbent for oxytetracycline adsorption
Source: Sci Rep. 2021 Mar 5;11:5276. doi: 10.1038/s41598-021-84231-8 (PMC7935931; doi:10.1038/s41598-021-84231-8)
Supplement: Supplementary file 1 — Supplementary Information. [file 41598_2021_84231_MOESM1_ESM.docx]

Supplementary files

Fig. S1 High resolution (a) Si, (b) Al and (c) Mn XPS spectra of GTS, Na500, Na700 and S500.
